# Executive Function and Transfer Effect Training in Children: A Behavioral and Event-Related Potential Pilot Study

**DOI:** 10.3390/bs15070956

**Published:** 2025-07-15

**Authors:** Chen Cheng, Baoxi Wang

**Affiliations:** School of Psychology, Jiangxi Normal University, Nanchang 330022, China; 202250000028@jxnu.edu.cn

**Keywords:** executive function training, updating, inhibition, near transfer, far transfer

## Abstract

This study examined the effect of executive function training targeting both updating and inhibition in children. The training included both single training (i.e., number 2-back training) and combined training (i.e., number 2-back and fish flanker training). Event-related potentials were also recorded. In Experiment 1, we employed both single-training and combined-training groups, which were contrasted with each other and with an active control group. In Experiment 2, the control group and the combined-training group were recruited to perform training tasks identical to those used in Experiment 1, and their EEG data were collected during the pretest and posttest stage. Experiment 1 found that the single group showed clear evidence for transfer to letter 2-back task compared with the active control group. The combined group showed significant transfer to the letter 2-back and arrow flanker task. Both groups found no transfer to fluid intelligence or shifting. Experiment 2 revealed that the participants who received updating and inhibition training showed a significant reduction in N2 amplitude and a significant increase in P300 amplitude after training in comparison to the active control group. Importantly, there was a significant positive correlation between reduced N2 amplitude and decreased response time in conflict effects. Additionally, there was a strong positive trend toward a relationship between behavioral performance improvement and an increase in P300 amplitude. From the perspective of the near-transfer effect, combined training is more effective than single training. Our results showed that the extent of transfer depends on the cognitive component overlap between the training and transfer tasks.

## 1. Introduction

Executive functions refer to a set of higher-level cognitive processes that help individuals sustain attention, address problems, pursue goals, and manage their behaviors, thoughts, and emotions (Diamond, 2013; Miyake et al., 2000). Typically, the executive functions comprise three components: (a) shifting between tasks, (b) updating and monitoring of working memory representations, and (c) inhibition of prepotent responses (Miyake et al., 2000; Zuber et al., 2023). Executive functions are reportedly related to a wide range of abilities in children, such as fluid intelligence (Sluis et al., 2007), mathematical achievement (Purpura et al., 2017), decision-making (P. Xu et al., 2020), or creativity (Cassotti et al., 2016). In consideration of the important effect of executive function on cognitive development and academic achievement, some studies have focused on executive function training in children (Dong et al., 2024; Menu et al., 2022). Executive function training commonly employs computerized training tasks such as the flanker task, which is used to measure inhibition capacity (Sari et al., 2016), the n-back task, which is used to measure updating capacity (Dong et al., 2024; Soveri et al., 2017), and the switching task in which the switching cost is measured by computing the difference between switch trials and repeat trials (Pereg et al., 2013).

Both near- and far-transfer effects are considered in studies of executive function training (Kassai et al., 2019). Near transfer refers to enhanced performance in a structurally similar task that involves the same cognitive ability as the training task (e.g., transfer of digit N-back to letter N-back), and far transfer refers to improvement in a structurally different task that involves different cognitive abilities from those engaged in the training task, such as executive function training, leading to better performance in a task measuring fluid intelligence (Soveri et al., 2017; Von Bastian & Oberauer, 2014). Although a recent meta-analysis focused on executive function training in children has indicated that the benefits of such training can be observed in both near- and far-transfer effects (Birtwistle et al., 2025), other meta-analyses suggest that a cautious attitude should still be maintained toward far-transfer effects (Sala & Gobet, 2017, 2020). For example, the findings of the meta-analysis conducted by Sala and Gobet (2017) revealed that the training yielded significant but small far-transfer effects (g = 0.12), as well as transfer effects on mathematical abilities (g = 0.20). However, it failed to effectively enhance the cognitive and academic skills of typically developing children. Another meta-analysis study also found that the intellectual performance and academic achievement (such as language and mathematical abilities) of typically developing children were largely unaffected by the training (g = 0.09). In particular, when an active control group was used in the studies, the effect size of far-transfer was close to zero (Sala & Gobet, 2020). Therefore, in exploring the transfer effects of executive function training, the type of the control group is a key variable. The observed experimental effect is vulnerable to being confounded by expectancy effects when using a no-contact control group (Melby-Lervåg & Hulme, 2016). The positive far transfer found in some training studies might be attributed to using a no-contact control group (Melby-Lervåg & Hulme, 2016; Schmiedek et al., 2010). In order to exclude this confounding variable, the current study used an active control group to increase the internal validity of the conclusions regarding the training program.

Childhood, a crucial period for the development of brain and cognitive functions, as indicated by Giedd et al. (2015), also serves as a pivotal stage for executive function training. Younger children may be more likely to benefit from training because of greater neuroplasticity (Best & Miller, 2010; Huizinga et al., 2006), but older children may benefit more because the neural underpinnings of executive function—prefrontal networks—continue to undergo important structural and synaptic changes throughout late childhood and adolescence (Best & Miller, 2010; Diamond, 2013). Relatedly, previous research has also shown that other important cognitive skills, such as metacognitive skills, develop progressively with schooling, and are better developed by the end of middle childhood (Schneider & Lockl, 2008; Schneider & Löffler, 2016). Improved metacognitive abilities contribute to learning in a range of school subjects (Dimmitt & McCormick, 2012; Smortchkova & Shea, 2020), and may also enhance the benefits of training for older children.

However, executive function training has mainly targeted a single component of executive function. For example, some studies have focused on inhibition training by using go/no-go, flanker, and Stroop tasks (Dong et al., 2024; Volckaert & Noël, 2015). Some studies have focused on working memory updating training by using n-back and running working memory tasks (X. Chen et al., 2018; Kubota et al., 2023). Compared with a large number of single-component executive function training studies, one study combined different types of executive function components in a training program (Röthlisberger et al., 2012). This training program focused mainly on working memory, interference control, and cognitive flexibility processes. The results revealed significant near-transfer effects in all three components of executive function. The study showed that the magnitude of the transfer effect depends on the degree of cognitive overlap between the transfer task and the training task. According to process-specific transfer hypothesis, cognitive training yields improvement on transfer tasks only to the extent that the transfer tasks share the same underlying cognitive processes with the training tasks (Dahlin et al., 2008; Sprenger et al., 2013). From this hypothesis, we could conclude that the breadth of the transfer effect should reflect the breadth of training.

Updating is widely recognized as the most critical aspect within executive functions (Juras et al., 2024). This is because it is involved in fluid reasoning processes and holds significant importance in solving daily tasks that require high cognitive abilities (T. Chen & Li, 2007; McAlister & Schmitter-Edgecombe, 2016). Furthermore, the association between inhibition and updating may be closer than previously thought. On the one hand, the inhibition mechanism may be embedded within the updating process and serve as a core component of it (Fleming et al., 2016). On the other hand, updating training can also enhance inhibitory control ability (Heinzel et al., 2016; Karbach & Verhaeghen, 2014). Integrated training that combines updating and inhibition may promote more extensive transfer effects (Karbach & Kray, 2016). Therefore, the combined training in this study incorporates 2-back training and flanker task training, aiming to investigate whether it can produce greater transfer effects compared to single training.

According to the Donders Subtraction Method (Danek & Mordkoff, 2011), we can test whether combined training produces the greater transfer effect relative to single training. More specifically, the control group was considered the baseline condition, which involved the common cognitive processes across single and combined-training conditions. The transfer effects from baseline condition are subtracted from that of the single training (2-back training) to reflect the effects of 2-back training. The transfer effects from the single-training condition are subtracted from that of the combined training (2-back and flanker training) to reflect the effects of flanker training. However, the previous combination training study had two limitations. One was using a no-contact control group that could result in an expectation effect. The other was lack of investigation to clarify the extent of the training effects to other untrained cognitive functions due to lack of a far transfer task. Therefore, it remains unclear whether combination training would lead to much greater transfer effects.

Since neural development is partly dependent on learning skills, the neural changes that occur during the natural development of working memory may share some commonalities with those brought about by training (Jones et al., 2022). As previously discovered in research, repeated and long-term working memory training can lead to neuroplastic changes, thereby supporting the enhancement of cognitive abilities (Klingberg, 2010). Therefore, it is important to understand the changes in neural mechanisms after training by utilizing Event-Related Potential (ERP) technology. The N2 and P300 components are closely related to executive functions. N2 has consistently been associated with inhibition processing in flanker tasks. It occurs in the window between 200 ms and 400 ms post-stimulus onset, and is thought to be generated in the frontocentral scalp locations (Xie et al., 2017). N2 amplitudes are larger for incongruent trials than for congruent trials in the flanker task, which is considered to reflect inhibition ability (Hoyniak & Petersen, 2019). One study found a significant reduction in N2 amplitude in a go/no-go task after 20 days of working memory training, suggesting improved inhibition ability (Wang et al., 2022).

The P300 component, which is closely related to context updating, was detected at 400–700 ms following stimulus onset, with a broad scalp distribution spanning the posterior areas (Donchin & Coles, 1988; Fields, 2023). One study used the running memory training task in healthy adult participants to explore the influence of updating–function training on brain activity with ERPs. The results showed that the P300 amplitudes increased significantly after training, which suggests that training improved the participants’ updating capacity (Zhao et al., 2013). A recent study has also found that there was a significant increase in P300 amplitude with n-back training in young adults, indicating that a large improvement in updating processes involved in the one-back task (Salmi et al., 2019). However, very few ERP studies have investigated the neural correlates of executive function training in children.

In summary, the current study aimed at examining the effect of executive function training targeting both updating and inhibition in children with two experiments. In Experiment 1, we employed both single-training and combined-training groups, which were contrasted with each other and with an active control group. Greater improvements were expected for the combined-training groups in both near-transfer and far-transfer tasks, given that the combined-training task could further contribute to cognitive enhancement. In Experiment 2, we aimed to investigate a potential neural basis of training-induced changes in executive function performance. Given that the N2 component reflects inhibition, it was expected that the N2 amplitudes would be reduced after inhibition training. Considering that the P300 reportedly reflects context updating, P300 amplitudes were expected to increase after the updating training.

## 2. Experiment 1

### 2.1. Methods

#### 2.1.1. Participants

Before the formal study, G*Power software (v. 3.1.9.2; Faul et al., 2007) was used to estimate the sample size needed for the study. Assuming a medium effect size *f* = 0.25, *α* = 0.05, power = 0.80, and two repeated measurements (estimated correlation 0), a minimum of 42 participants (14 in each group) would be required to detect significant intra- and intergroup interaction effects in a repeated-measure ANOVA.

Fifty-eight fifth-grade primary school students from China participated in Experiment 1. The exclusion criteria for all participants were as follows: (1) caregiver—reported visual impairment that could not be adequately corrected with the use of glasses; (2) hearing impairment that remained uncorrected despite the application of a hearing aid; (3) fine motor impairment; (4) and/or intellectual disability. These conditions were deemed likely to impede the participants’ capacity to engage fully in both the testing procedures and the intervention components of the research. The parents of all the children gave written consent to be involved in this experiment. All participants were included in the final analysis. The children were randomly assigned to three age groups: the active control group (*n* = 20, 9 girls, 11 boys, mean = 128 months, *SD* = 6 months, range 10 to 12 years); the single-training group *(n* = 20, 11 girls, 9 boys, mean = 130 months, *SD* = 7 months, range 9 to 12 years); and the combined-training group (*n* = 18, 11 girls, 7 boys, mean = 128 months, *SD* = 7 months, range 9 to 12 years). All participants were healthy, right-handed, had normal or corrected-to-normal vision, and had no history and current symptoms of affective disorders. Participants received a small gift when they completed each training session. This study has been approved by the Ethical Committee of the University (approval number: JXNU-PSY-2024022).

#### 2.1.2. Pre- and Post-Training Test Tasks

##### Number 2-Back Task

This task was used to measure the updating of working memory (Zhao et al., 2018). A single digit consisting of numbers 1–9 was presented serially in the center of a computer screen. The presentation color of each task stimulus was white, and its point size was 60. The participants were asked to press the C key when the digit was the same as that viewed two digits ago back. The participant pressed the M key when the digit was not the same as that viewed two digits ago. Each digit was presented for 500 ms, followed by a blank screen for 3000 ms, and the participants had 3500 ms to make a response. A total of 80 trials consisted of two blocks of 40 trials each. Additionally, 50% of the trials were match trials. According to the signal detection theory, the d′ was used as a measure of sensitivity (d′ = Z (hit rate) − Z (false alarm rate)) (Ortiz-Rios et al., 2023), which was regarded as a useful metric for an n-back task because it measures the hit rate while penalizing for false alarms. A lower d′ score indicates poorer working memory updating performance (Haatveit et al., 2010).

##### Arrow Flanker Task

This task consisted of a centrally presented target flanked by two flanker arrows on each side. In congruent trials, the flanker arrow was the same as the target arrow (for example, <<<<<); on incongruent trials, the flanker arrow was in the opposite direction of the target (for example, < < > < <). Each task stimulus was rendered in white with a size of 32. The participants were instructed to press the F key with the left hand if the target was ‘<’, and to press the J key with the right hand if the target was ‘>’, while ignoring the flanks (Eriksen & Eriksen, 1974). Each subject completed two blocks, each consisting of 80 trials. The blocks were presented in a random order; 50% of the blocks consisted of congruent condition trials, and 50% consisted of incongruent trials. The stimuli were presented using the E-Prime 2.0 software package. Based on the previous flanker study conducted on this age group (Johnstone & Galletta, 2013), the stimulus was presented with a fixation for 500 ms, a blank screen for 500 ms, and the target for 1500 ms or until the participant responded. The response–stimulus interval was 1500 ms.

##### Number Switching Task

The number switching task is a widely used switching task which is used to measure the ability to flexibly switch between tasks (Rogers & Monsell, 1995). A series of stimuli (the digits ‘0–9’, but digit ‘5’) were presented sequentially at the center of the screen. The participants were asked to perform three sets of tasks. The first rule for the task was to determine whether the digit was more than 5 or less than 5 (more–less). The participants were instructed to press the S key with the left hand when the digit was less than 5, and to press the L key with the right hand when the digit was more than 5. The second rule was to determine whether the digit was even or odd (even-odd). The participants were instructed to press the S key with the left hand when the digit was odd, and to press the L key with the right hand when the digit was even. The third rule was to switch according to the background color of the digit. If the background color was blue, the participants performed the more–less task, and if the background color was red, the participants performed the odd even task. The same digits were never repeated in the successive trials (Salthouse et al., 1998). The presentation color of each task stimulus was black, and its point size was 18. Each participant completed two blocks, with 32 trials in each block; 50% of the blocks consisted of switching trials, and 50% of the blocks consisted of non-switch trials. The digits were presented for 1500 ms or until the participant responded, and the next stimulus was presented immediately after the participant’s response. The switch cost was computed by subtracting the time of the non-switch trials from the time of the switch trials (Rueda et al., 2012, 2004).

##### Raven’s Advanced Progressive Matrix Test

Raven’s standard progressive matrix (SPM) test was used to measure the nonverbal fluid intelligence (Raven et al., 2000). The standard Chinese version of the test was used in this study. The participants were required to complete the test in 40 min. The dependent measure was the number of correctly solved problems.

#### 2.1.3. Training Task

##### Letter 2-Back Task

The letter 2-back task was consistent with the number 2-back task, except that the stimuli were English letters. The participants received feedback on the screen about the percentage of correct responses after finishing each block. The procedure took around 40 min in the single-training group, and 20 min in the combined-training group. The d prime (d′ = Z (hit rate) − Z (false alarm rate)) was used as a measure of training performance for each session.

##### Fish Flanker Task

The fish flanker task was identical to the arrow flanker task, except that five colorful fish replaced the arrows (Rueda et al., 2004). Participants were asked to press a key depending on the direction in which the middle fish was swimming while ignoring the flanker fish. The participants were informed that if the middle fish swam to the “left”, they should press the F key with their left hand; if the middle fish swam to the “right”, they should press the J key with their right hand. Each participant completed two blocks, each consisting of 80 trials; 50% of the blocks consisted of congruent condition trials, and 50% consisted of incongruent trials. The stimuli were presented with a fixation for 500 ms, a blank screen for 500 ms, and the target for 1500 ms or until the participant responded. The response–stimulus interval was 1500 ms. The participants received feedback on the screen about the percentage of correct responses and RT after finishing each block. Based on the previous literature (Zhao & Jia, 2019; Pronk et al., 2023), the difference in RT was used as a measure of inhibition ability, which is computed by subtracting the mean RT on congruent trials from the mean RT on incongruent trials. This score was used as the dependent variable, with a low score indicating stronger inhibition control (Zhao & Jia, 2019).

#### 2.1.4. Procedures

Each child completed a battery of number 2-back, arrow flanker, switching and Raven’s standard progressive matrix task before, immediately after, and three months after training. Two trained groups completed ten 40 min sessions within five weeks (2 sessions per week). The single-training group performed the letter 2-back task, whereas the combined-training group completed both the letter 2-back task and fish flanker task. Two training tasks were not adaptive, but participants were given feedback about their accuracy and response time (RT) after each training session. The participants were asked to improve accuracy and RT as soon as possible.

The active control group learned to perform the Chinese input method, and the basic operation of Word and Excel for the same amount of time in the computer classroom as the two training groups’ sessions. The content and nature of these activities were specifically chosen, and they involved some basic and simple operations.

### 2.2. Statistical Analysis

All data analyses were conducted in SPSS 23.0. For dependent variables involving multiple groups, a repeated-measure analysis of variance (ANOVA) was conducted, with “group” as the between-subject variable and “session” as the within-subject variable. For dependent variables that only involved a single group of training, a paired sample t-test was used to analyze the training sessions. In the analysis of the training phase, “session” included two levels, which were the first training session (i.e., session 1) and the last training session (i.e., session 10). In the analysis of the transfer effects, the two levels included in “session” were the pretest and posttest of the testing task, respectively. For all analyses, the significance criterion was set to 0.05. Partial η-squared values (η^2^) were used to ascertain the effect sizes for the ANOVA. Post hoc analyses with Bonferroni correction were conducted.

### 2.3. Results

#### 2.3.1. Training Results

##### Letter 2-Back Task

**Figure 1:**
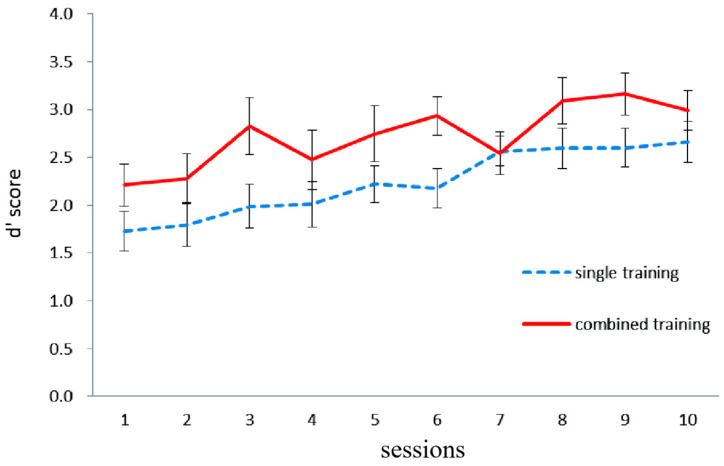
The working memory updating performance (dependent variable: d′ score) in both single and combined groups on each of the 10 letter 2-back training sessions in Experiment 1. The d′ score for updating performance on the letter 2-back task is the Z-score of correctly detected hits minus the Z-score of false alarms on non-hit trials. Error bars represent standard deviations.

Figure 1 illustrates working memory updating performance in the letter 2-back task in both the single and combined groups. The mean training performance during the first and last session was taken as the dependent variable. A 2-group (single, combined) × 2 session (first, last) mixed-design ANOVA was conducted, with the group as the between-subject factor and the session as the within-subject factor. The main effect of the session was significant, *F* (1, 36) = 33.04, *p* < 0.0001, η^2^ = 0.48, indicating that the training performance in the last session was significantly higher than that in the first session. The main effect of the group was not significant, *F* (1, 36) = 2.48, *p* = 0.12, and the interaction effect between the test time and group was not significant, *F* (1, 36) = 0.26, *p* = 0.61.

##### Fish Flanker Task

**Figure 2:**
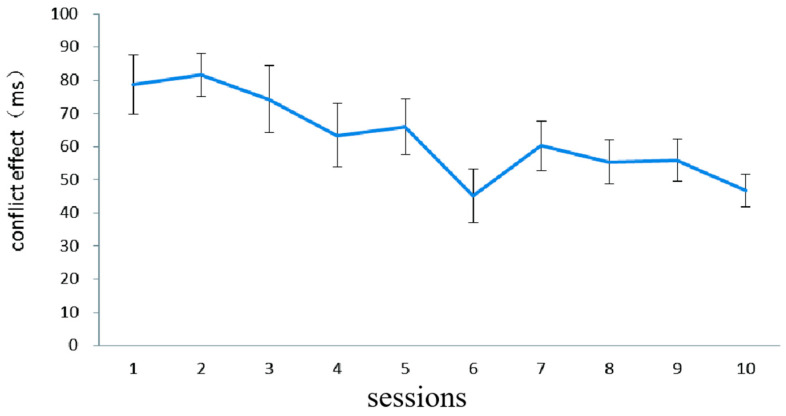
The inhibition control performance (dependent variable: conflict effect) in the combined group on each of the 10 fish flanker task training sessions in Experiment 1. The conflict effect was computed by subtracting the mean RT on congruent trials from the mean RT on incongruent trials. Error bars represent standard deviations.

Figure 2 illustrates the difference in RT (i.e., conflict effect) in the fish flanker task in the combined group. In order to investigate whether there was any improvement after training, the paired sample *t*-test was conducted on the RT data from the first session and the last session. There was a significant improvement, *t* (17) = 3.59, *p* < 0.01, indicating that the inhibition control capability of the combined-training group was improved by the fish flanker training.

#### 2.3.2. Near Transfer Results

##### Number 2-Back

**Figure 3:**
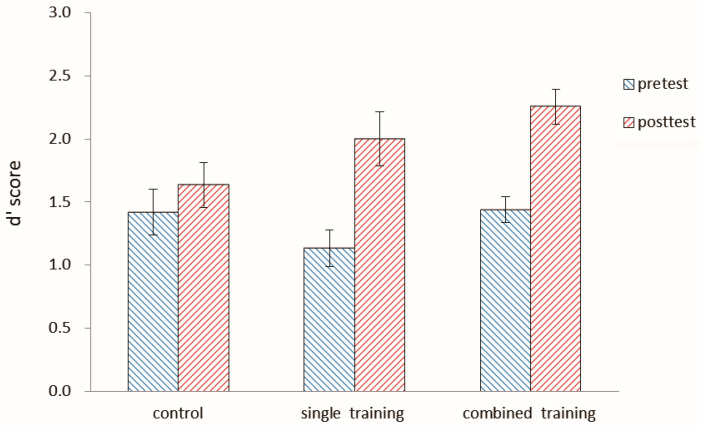
The d′ score of both the pretest and posttest for the number 2-back in the three groups in Experiment 1. The d′ score is the Z-score of correctly detected hits minus the Z-score of false alarms on non-hit trials. Error bars represent standard deviations. *Note*: The pretest was conducted one week before the first training session, and the posttest was administered one week after the last training session.

Both pretest and posttest d’ scores for the number 2-back task in the three groups are shown in Figure 3 and Table 1. In order to examine the effect of the letter 2-back training on the near transfer task, a 3-group (control, single, combined) × 2 session (pretest, posttest) ANOVA was conducted. There was a main effect of the session, *F* (1, 55) = 37.28, *p* < 0.0001, η^2^ = 0.40, and a significant interaction between the session and group, *F* (2, 55) = 4.22, *p* < 0.05, η^2^ = 0.13. The simple effect of the session within the group showed a significantly higher performance posttest than pretest in both the single group, *F* (1, 55) = 24.26, *p* < 0.05, η^2^ = 0.31, and the combined group, *F* (1, 55) = 19.35, *p* < 0.05, η^2^ = 0.26; however, there was no significant difference between pretest and posttest scores in the control group, *F* (1, 55) = 1.46, *p* = 0.23. The effect size was measured by Cohen’s d for comparison of the training effects for each group (J. D. Cohen, 1992). There was a large effect for both the single group (d = 1.06) and the combined group (d = 1.58), but a small effect for the control group (d = 0.26). The results showed that the updating training of executive functions produced significant near-transfer effect.

##### Arrow Flanker Task

Table 2 summarizes the mean reaction times for congruent and incongruent trials on the arrow flanker task in the three groups at pretest and posttest. In order to investigate the effect of fish flanker task training on inhibition ability, we ran a 2-session (pretest; posttest) × 3 group (control, single, combined) ANOVA with the conflict effects (as measured by incongruent–congruent difference on RT) as the dependent variable. The main effect of the session was significant, *F* (1, 55) = 43.55, *p* < 0.0001, η^2^ = 0.44, indicating that the difference on the RT of posttest was less than that of the pretest. There was no significant main effect of group, *F* (2, 55) = 2.62, *p* > 0.05, and no significant interaction between the session and the group, *F* (2, 55) = 2.01, *p* > 0.05.

We used the effect size as a reference index. The combined group showed a relatively larger training effect (d = 1.13) compared with the single group (d = 0.8) and the control group (d = 0.58). In summary, these results indicate that the fish flanker task training produced a significant near transfer effect in the combined group.

#### 2.3.3. Far Transfer Results on Both the Switching Task and Fluid Intelligence

##### Switching Task

Table 3 presents the mean reaction times and accuracy for repeating and switching trials for the three groups at pretest and posttest. In the switching task, the dependent variable of the task was the switching cost. To examine the far-transfer effect in this task, we ran a 2 (test time: pretest, posttest) × 3 (group: control, single, combined) ANOVA. There was no significant main effect of the test time, *F* (1, 55) = 0.19, *p* = 0.67, the main effect of the group, *F* (2, 55) = 0.85, *p* = 0.43, and the interaction between the test time and the group, *F* (2, 55) = 0.85, *p* > 0.05. The results showed that there was no far-transfer effect in either the single group or the combined group.

##### Fluid Intelligence

Based on the revised Chinese-version urban children’s norms, the SPM test raw scores of each participant were converted into standard Z-scores for analysis. Table 4 presents the mean of the Z-scores for the three groups. A 3 (test time: pretest, immediate posttest, tracking test) × 3 (group: control, single, combined) ANOVA was performed. The results showed that there was no significant main effect of the test time, *F* (2, 110) = 1.62, *p* = 0.20, the main effect of group, *F* (2, 55) = 0.02, *p* = 0.98, and the interaction between the test time and the group, *F* (2, 110) = 0.76, *p* = 0.55. The results indicate that the executive function training did not improve fluid intelligence.

### 2.4. Discussion

The results of Experiment 1 show that the combined group targeting both the updating and inhibition components produced clear near-transfer effects for both the number 2-back task and the arrow flanker task. The single-group targeting updating component only produced near transfer to the number 2-back task, while the control group did not produce any near-transfer effects. According to the subtractive method, we concluded that 2-back training contributed to subsequent updating enhancement by subtracting the control group from the single group, and that flanker training contributed to subsequent inhibition improvement. We found no specific evidence of far transfer to fluid intelligence for any one training group. Inconsistent with our prediction, the combined group did not show the largest numerical gains, and only produced near-transfer effects. Considering the independent contribution of each component training task to cognitive improvement in the combined group, we further explored the neural mechanism of training enhancement by employing the combined and control groups in Experiment 2.

## 3. Experiment 2

### 3.1. Methods

#### 3.1.1. Participants

Participants included 39 students from the fifth grade of a primary school in China. All experimental participation criteria were consistent with Experiment 1. Data from three participants in the combined-training group were excluded due to excessive artifacts caused by head movements, resulting in a final total sample of 36 participants. Participants were divided into two groups: the control group (*n* = 20, 9 girls, 11 boys, mean = 135 months, *SD* = 9 months, range 10 to 12 years); and the combined-training group (*n* = 16, 9 girls, 7 boys, mean = 133 months, *SD* = 7 months, range 10 to 12 years).

The sample size adequacy was evaluated using G*Power for post hoc power analyses (Faul et al., 2009). Given that we obtained 36 valid samples, the results of the post hoc analysis revealed a power of 0.83 (>0.80) with small effect sizes (*f* = 0.25), which indicated that our final valid sample possessed sufficient power (J. Cohen, 2013).

All participants were healthy, right-handed, had normal or corrected-to-normal vision, and had no history and current symptoms of affective disorder. All parents agreed to allow their children to take part in the experiment and signed informed consent. At the end of the experiment, the participants were given a gift.

#### 3.1.2. Training Tasks and Procedures

The training sessions of executive function were identical to those used in Experiment 1. We examined only the near-transfer effect and its related neural mechanisms.

#### 3.1.3. EEG Recording and Analysis

Electroencephalograms (EEGs) were recorded using a Neuroscan 32-channel system, following the standard procedures outlined in a previous study on a sample of children (J. Xu et al., 2021). Ag-AgCl electrodes were mounted on an elastic cap according to the 10–20 system of electrode placement. All channels were referenced to an electrode placed on the left mastoid during recording. Electrooculograms (EOGs) were recorded from electrodes placed above and below the left eye (vertical EOG), and on the outer canthus of both eyes (horizontal EOG). All electrode impedances were kept below 5 KΩ. EEG and EOG signals were amplified using a DC-100 Hz band-pass filter, and the sampling rate was 500 Hz.

EEG data were re-referenced to the binary linked-mastoid reference electrodes offline by subtracting half of the amplitudes recorded at the right mastoid from all data recorded in each channel. Trials with large eye or body movements were rejected via manual editing. Trials with an amplitude exceeding ±100 µV were rejected to remove the artifacts induced by eye movements, muscle artifacts, electrode drifting, or other artifacts. And trials in which participants made response errors were excluded from analysis. Ocular artefacts were corrected automatically with an eye movement correction algorithm. Based on the previous literature (Kim et al., 2024; Torres et al., 2024; Zarantonello et al., 2024), EEG data were filtered offline with a high-pass filter of 0.01 Hz and a low-pass filter of 30 Hz. The continuous EEG data were segmented into 1000 ms epochs, beginning 200 ms prior to the onset of stimulus, and were averaged separately for each participant and each condition. In the flanker task, the number of analyzed trials ranged from 33 to 77 under the incongruent condition, and from 31 to 76 under the congruent condition. In the 2-back task, the number of analyzed trials ranged from 27 to 74. More specifically, for the flanker task, the mean numbers of analyzed trials were 69.8 (congruent) and 70 (incongruent) for the control group, and 66.3 (congruent) and 65.5 (incongruent) for the combined group during the pretest stage; and 64.7 (congruent) and 68.3 (incongruent) for the control group, and 54.3 (congruent) and 55.2 (incongruent) for the combined group during the posttest stage. For the 2-back task, the mean numbers of analyzed trials were 66.5 (pretest) and 61.6 (posttest) for the control group, and 62.8 (pretest) and 62.6 (posttest) for the combined group.

For the flanker task, the mean amplitudes of N2 were measured from grand-averaged ERPs during the window between 350 and 450 ms after stimulus onset (Checa et al., 2014; Hermansen et al., 2017). For the 2-back task, the amplitude of P300 was measured as mean voltages between 400 ms and 600 ms after target stimulus onset (Covey et al., 2019; Guo et al., 2023). The analyses of N2 and P300 included five electrodes (Fz, FCz, Cz, CPz, Pz). These electrodes were selected because the amplitude of both N2 and P300 was demonstrated to be maximal by the observation of distribution of both components in the current study. In addition, these regions reflected the effect of conflict processing in the flanker task and updating context in 2-back task in previous studies (Dahlin et al., 2008). In the event that the data exhibited a normal distribution, we further employed Pearson’s correlation analysis to assess the degree of correlation between the behavioral measures (RT, d′) and the electrophysiological components (N2, P300). Conversely, if the data did not follow a normal distribution, Spearman’s rank correlation was employed to explore the degree of correlation between them.

#### 3.1.4. Behavioral Data Analysis

Behavioral data were analyzed using the same method as in Experiment 1.

### 3.2. Behavior Results

#### 3.2.1. Training Results

##### Letter 2-Back Task

**Figure 4:**
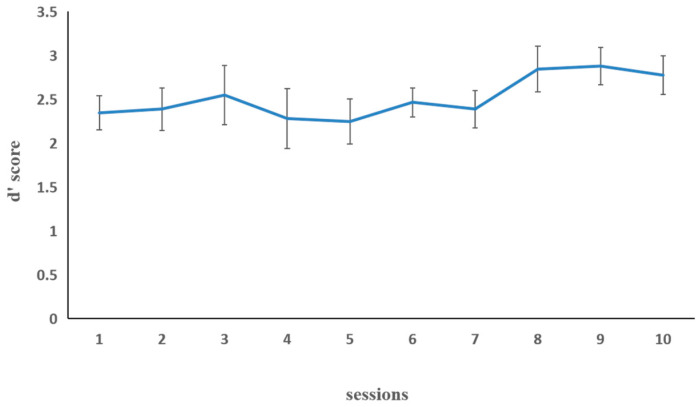
The working memory updating performance (dependent variable: d′ score) in the combined group for the each of the 10 letter 2-back training sessions in Experiment 2. The d′ score is the Z-score of correctly detected hits minus the Z-score of false alarms on non-hit trials. Error bars represent standard deviations.

Figure 4 illustrates working memory updating performance in the letter 2-back task in the combined group. The mean training performance (d′ score) during the first and last sessions was taken as the dependent variable. The paired sample t-test on d′ score showed a significant improvement, *t* (15) = −4.46, *p* < 0.001, indicating that the training performance of the last session (*M* = 2.92, *SD* = 0.35) was significantly higher than that of the first session (*M* = 2.31, *SD* = 0.50).

##### Fish Flanker Task

**Figure 5:**
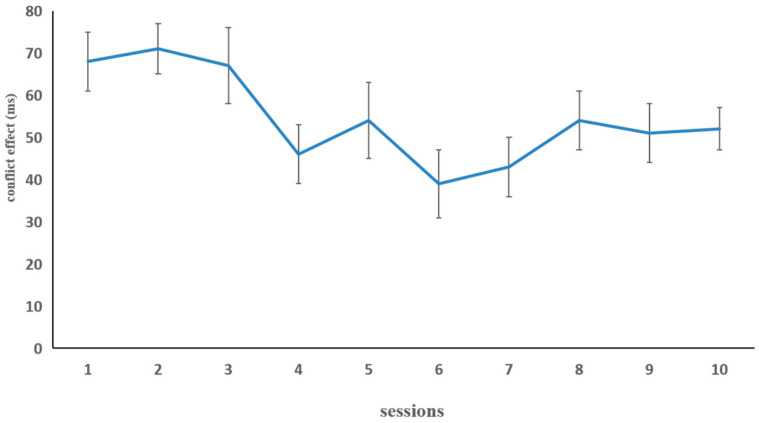
The inhibition control performance (dependent variable: conflict effect) in the combined group for each of the 10 fish flanker task training sessions in Experiment 2. The conflict effect was computed by subtracting the mean RT on congruent trials from the mean RT on incongruent trials. Error bars represent standard deviations.

Figure 5 illustrates the difference in RTs in the fish flanker task in the combined group. For the fish flanker task, we conducted a paired sample t-test using the reaction times (RTs) in the first session and the last session as the data for comparison. There was a significant improvement, *t* (15) = 2.54, *p* < 0.05, indicating that the inhibition control capability of the combined-training group was improved by the fish flanker training.

#### 3.2.2. Near Transfer Results

##### Number 2-Back Task

**Figure 6:**
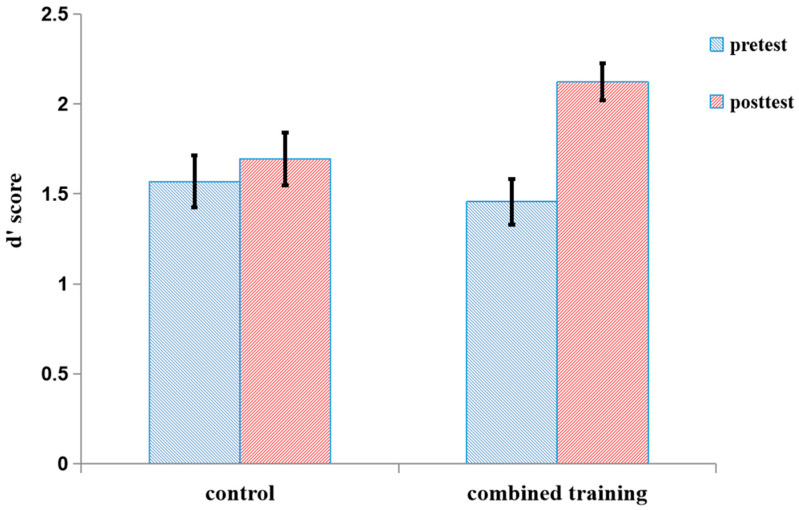
The d′ score of both the pretest and posttest for the number 2-back for the two groups in Experiment 2. The d′ score on the number 2-back is the Z-score of correctly detected hits minus the Z-score of false alarms on non-hit trials. Error bars represent standard deviations.

The d′ scores of both the pretest and posttest on the number 2-back task in the two groups are shown in Figure 6 and Table 5. For the number 2-back task, we conducted 2 group (training, control) × 2 session (pretest, posttest) ANOVA with the d′ score as the dependent variable. There was no significant main effect of the group, *F* (1, 34) = 1.06, *p* = 0.31. There was a main effect of the session, *F* (1, 34) = 13.02, *p* < 0.001, η^2^ = 0.28, and a significant interaction between the session and the group, *F* (1, 34) = 6.05, *p* < 0.05, η^2^ = 0.15. The simple effect of group within session showed significantly higher performance at posttest than at pretest for the training group, *F* (1, 34) = 16.56, *p* < 0.001, η^2^ = 0.33; however, there was no significant difference between the pretest and posttest for the control group, *F* (1, 34) = 0.74, *p* = 0.40. Furthermore, we used effect size as a reference index. There was a large effect for the training group (d = 1.31), but a small effect for the control group (d = 0.21).

##### Arrow Flanker Task

Table 6 summarizes the mean reaction times and accuracy for congruent and incongruent trials on the arrow flanker task for the two groups at pretest and posttest. For the arrow flanker task, we conducted a 2-group (training, control) × 2 session (pretest, posttest) ANOVA with the conflict effects (as measured by incongruent–congruent difference on RT) as the dependent variable. There was a main effect of the session, *F* (1, 34) = 26.25, *p* < 0.0001, η^2^ = 0.44, and a significant interaction between the session and the group, *F* (1, 34) = 3.99, *p* = 0.05, η^2^ = 0.11. There was no significant main effect of the group, *F* (1, 34) = 0.58, *p* = 0.45. The simple effect of session within the group showed significantly smaller conflict effects at posttest than at pretest for both the control group, *F* (1, 34) = 5.50, *p* = 0.03, η^2^ = 0.14 and the training group, *F* (1, 34) = 22.82, *p* < 0.001, η^2^ = 0.40. Further analysis used effect size, showing that there was a relatively larger training effect for the training group (d = 2.45) compared with the control group (d = 1.25).

In summary, the behavioral results of Experiment 2 repeated the model of Experiment 1. In the 2-back task, the combined-training group exhibited a significant improvement in the d′ score during the posttest. This result suggests that the combined-training regimen effectively enhanced the participants’ ability to update and maintain information in working memory. Moreover, in the flanker task assessing inhibitory control, the difference in response time of the combined-training group was significantly smaller compared to that of the control group. This indicates that the combined training not only improved updating ability, but also enhanced the participants’ inhibitory control ability, enabling them to better suppress irrelevant information.

### 3.3. ERP Results

N2 Amplitude

**Figure 7:**
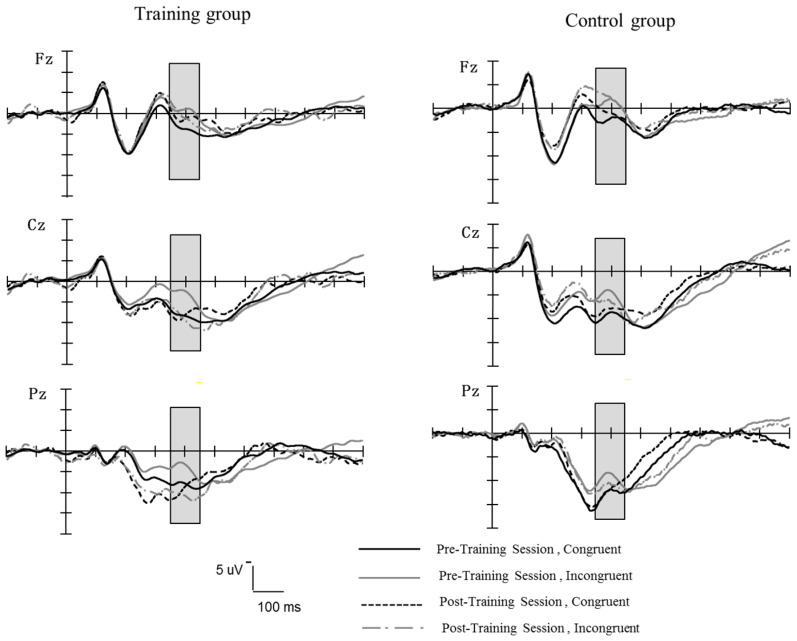
The grand-averaged waveforms of the N2 components elicited at the middle electrodes sites (Fz, Cz, Pz) for the training group and control group during the pretest and posttest stages in the flanker task.

Figure 7 shows the grand-averaged waveforms elicited at the middle electrode sites (Fz, Cz, Pz) for the training group and control group during the pretest and posttest stage. A repeated ANOVA on the mean amplitude (between 350 and 450 ms) of N2 was performed with the group (training, control) as the between-subject variable, and session (pretest, posttest), trial type (congruent, incongruent), and electrodes (Fz, FCz, Cz, CPz, Pz) were the within-subject factors. There was a significant main effect of trial type, *F* (1, 34) = 15.99, *p* < 0.0001, η^2^ = 0.32, and a significant interaction between the trial type and session, *F* (1, 34) = 8.33, *p* < 0.01, η^2^ = 0.20, and a significant interaction between the trial type, session, and group, *F* (1, 34) = 7.81, *p* < 0.01, η^2^ = 0.19. Further simple effect analysis showed that (1) in the control group, incongruent trials elicited a significantly larger N2 amplitude than congruent trials at both pretest and posttest (pretest, *F* (1, 34) = 12.79, *p* < 0.001, η^2^ = 0.27, posttest, *F* (1, 34) = 7.19, *p* = 0.01, η^2^ = 0.17, respectively); (2) in the training group, incongruent trials elicited a significantly larger average N2 amplitude than congruent trials at pretest, *F* (1, 34) = 20.01, *p* < 0.0001, η^2^ = 0.37. However, at posttest, there was no significant difference in N2 amplitude between congruent and incongruent trials, *F* (1, 34) = 0.28, *p* = 0.61. The main effect of the group and other interactions concerning the group was not significant (*p*s > 0.05).

P300 Amplitude

**Figure 8:**
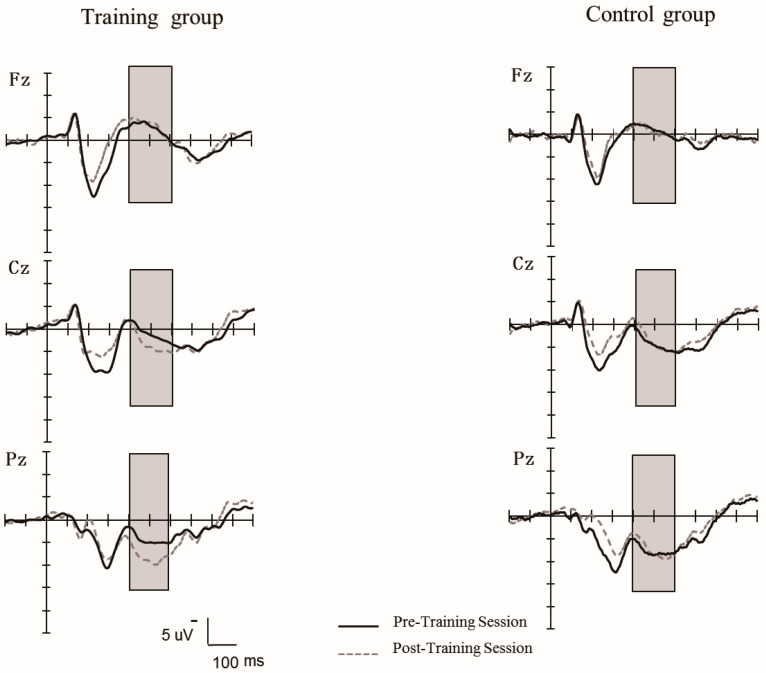
The grand-averaged waveforms of P300 components elicited at the middle electrodes sites (Fz, Cz, Pz) for the training group and control group during the pretest and posttest stages in 2-back task.

Figure 8 shows that the grand-averaged waveforms (P300) elicited at the middle electrode sites (Fz, Cz, Pz) for the training group and the control group during the pretest and posttest stage. A repeated ANOVA was performed with the group (training, control) as the between-subject variable and session (pretest, posttest), and the electrodes (Fz, FCz, Cz, CPz, Pz) were the within-subject factors. There was a significant main effect of electrodes, *F* (4, 136) = 73.64, *p* < 0.0001, η^2^ = 0.68, and a significant interaction between electrodes and session, *F* (4, 136) = 2.96, *p* < 0.05, η^2^ = 0.08, and a significant interaction between electrodes, session, and group, *F* (4, 136) = 5.07, *p* < 0.01, η^2^ = 0.13. Further simple effect analysis showed that (1) in the training group, there was larger P300 amplitude at posttest than at pretest only at Pz, *F* (1, 34) = 8.14, *p* < 0.01, η^2^ = 0.19; however, at other middle electrodes, there was no significant difference in P300 amplitude between pretest and posttest (*p*s > 0.05); (2) in the control group, no significant differences in P300 amplitude were found between pretest and posttest at each electrode. The main effect of the session and group, and other interactions was not significant (*p*s > 0.05).

### 3.4. Correlation Analysis

The Shapiro–Wilk test was used for testing normality. It was found that both the behavioral measures (RT, d′) and the electrophysiological components (N2, P300) followed a normal distribution. Therefore, Pearson’s correlation analysis was employed to explore the correlation between the behavioral results and ERP results.

Conflict effects refer to the difference between congruent and incongruent conditions, and they can be measured using both RT and N2 amplitude. The reduction in conflict effect for RT (as measured by posttest–pretest) was significantly associated with the reduction in conflict effect for N2 amplitude at Fz (as measured by posttest–pretest) in the training group, *r* (16) = 0.51, *p* < 0.05. This analysis reveals that as conflict resolution increased after training, the N2 amplitude decreased.

For the 2-back task, the difference in P300 amplitude between the pre- and posttest at Pz was marginally associated with the improvement in d′ between the pre- and posttest in the training group, *r* (16) = 0.44, *p* = 0.09. There was a strong tendency toward a relationship between updating improvement and the increase in P300 amplitude.

## 4. Discussion

Although previous studies on executive functions have identified the intervention effects of single training on children (Dong et al., 2024; Volckaert & Noël, 2015; Kubota et al., 2023), the effects of combined training as well as the changes in neural mechanisms post-training remain uncertain. Therefore, the present study examined the transfer effect of executive function training and related neural mechanisms. In Experiment 1, the single-training group, which received number 2-back training, showed clear evidence for transfer to a letter 2-back task compared to the active control group. The combined group, which received number 2-back and fish flanker task training, showed significant transfer to letter 2-back and arrow flanker tasks. Inconsistent with our prediction, in the combined-training group, no greater near- and far-transfer effects were produced with maximal efficiency. We further explored the neural mechanisms of training enhancement by employing a combined-training group in Experiment 2. The behavioral results of Experiment 2 repeated the model of Experiment 1. ERP analysis revealed that the participants in the combined-training group who received updating and inhibition training showed a significant reduction in N2 amplitude and a significant increase in P300 amplitude in the posttest. These results showed that the participants had enhanced conflict control and improved updating capacity after undergoing updating and inhibition training.

In terms of behavioral data, both the single-training group and the combined group showed a significant increase in d′ scores on the number n-back training task after completing the task. After the fish flanker training, the combined group exhibited a reduction in the conflict effect (i.e., subtracting the mean reaction time (RT) on congruent trials from the mean RT on incongruent trials) on this task. Importantly, the benefits of these trainings transferred to similar tasks, resulting in near-transfer effects. Compared to the control group, the single-training group demonstrated the transfer of training gains from the number n-back task to the letter 2-back task, performing better in the posttest than in the pretest (i.e., achieving higher d′ scores in the posttest). This is consistent with the findings of previous studies (Minear et al., 2016; Soveri et al., 2017). The findings from the meta-analysis reveal that following n-back training, there exists a moderate transfer effect to untrained n-back tasks (Soveri et al., 2017). In addition, compared to the single-training group, the combined-training group not only demonstrated near-transfer effects on the letter 2-back task, but also transferred the training benefits gained from the fish flanker task to the arrow flanker task. In the combined-training group, as the training on the fish flanker task progressed, the conflict effect decreased. This suggests that the participants gradually learned to disregard the interfering information brought about by the identity of the visual stimulus, and to inhibit the incorrect prepotent response associated with it, thereby generating the correct response required for the task (Stahl et al., 2014). In other words, after the fish flanker training, the combined-training group demonstrated improved inhibitory control ability, which transferred to the similar arrow flanker task.

Notably, in Experiment 2, the results of event-related potentials (ERPs) revealed neural mechanism changes that were consistent with the behavioral results. Specifically, the results of Experiment 2 showed a significant reduction in N2 amplitude for the flanker tasks in the combined-training group relative to the control group. Previous studies have shown that N2 was thought to be associated with the inhibition of irrelevant stimuli (Chueh et al., 2023; Heil et al., 2000). The current results reveal that flanker training improved the ability of inhibition processing in the combined group, as supported by the significant correlation between the reduced N2 amplitude and the decreased RT in conflict effects. The present findings are consistent with those of previous studies on cognitive control training (Millner et al., 2012). Millner et al. (2012) used cognitive control training tasks involving the Simon task and a go/no-go task in 3 consecutive days, and found greater reductions in RT interference effects and the largest reductions in the N2 amplitudes in a transfer task, suggesting that brief training can improve the ability to inhibit task-irrelevant information.

As in previous studies (Guo et al., 2023; Pergher et al., 2018; Tusch et al., 2016), the results of Experiment 2 also showed that the P300 amplitudes were enhanced after 2-back training. The P300 amplitude is considered a brain activity index of working memory updating (Fields, 2023). A recent study has found a significant increase in P300 amplitude with audiovisual n-back training, which indicates a large improvement in updating processes involved in the n-back task (Guo et al., 2023). Previous studies found that individuals were inclined to have a greater P300 amplitude at Pz (Gevins & Smith, 2000; Gongora et al., 2021). Our results found a strong positive trend toward a relationship between behavioral performance improvement and an increase in P300 amplitude at Pz in 2-back task in the training group, suggesting that the training could improve the function of working memory updating.

However, no greater far transfer was observed in the combined-training group. Although this does not align with the research hypothesis, it is generally consistent with the findings of some previous studies (Kassai et al., 2019; Minear et al., 2016; Zuber et al., 2023). For example, one training study targeting the updating component only found clear evidence for near transfer from spatial n-back training to other forms of n-back training, but did not find a far-transfer effect on measures of speed of processing, working memory, and reasoning (Minear et al., 2016). It is important to note that one possible reason for the lack of far transfer to fluid intelligence in our study is the use of active control groups (Melby-Lervåg & Hulme, 2013). Some of the studies that have found far-transfer effects on working memory updating training did not employ active control groups, which might signify that the far-transfer effect is attributable to an expectation effect (Klingberg, 2010; Shipstead et al., 2012).

Furthermore, the lack of such a far-transfer effect may also be related to the specialization of executive functions with age, whereby the uniformity of executive functions decreases and their diversity increases, leading to decreased common shared executive functions. Although updating, shifting, and inhibition may rely on similar underlying cognitive processes in childhood, executive functions become more specialized and independent with age (Hartung et al., 2020; F. Xu et al., 2013). This is supported by a previous brain imaging study that reported increasing segregation of structural brain network modules with age, and that this segregation mediates the effects of age on executive function (Baum et al., 2017). Therefore, as fifth graders are in the later stages of childhood, the lack of far-transfer effects in this study may be partly due to the specialization and specificity shown in their executive functioning.

The behavioral findings of a near-transfer effect could be interpreted by the process-specific hypothesis. This hypothesis considers that cognitive training yields improvement in transfer tasks only to the extent that the transfer tasks share the same underlying cognitive processes with the training tasks (Dahlin et al., 2008; Sprenger et al., 2013). The behavioral results provided evidence that training produced improvements only on tasks that shared a similar component with the training tasks, but no evidence that the training enhancement extended to tasks involving components other than those targeted by the training tasks. For example, participants in the single group, who trained with the number 2-back task, had enhanced performance on the letter 2-back task, but not on the flanker task, task switching, and fluid intelligence. Participants in the combined group, who trained with the number 2-back and fish flanker tasks, had improved performance on the letter 2-back and arrow flanker tasks, but no gain in the switching task and fluid intelligence. The present finding supports the hypothesis that the effects of training are task-specific. The finding that training a specific component of executive function leads to transfer on the specific component of executive function only suggests that the magnitude of transfer effects depends on the cognitive overlap between the transfer tasks and the training task (Soveri et al., 2017).

Although the current study provides important insights into the effectiveness of executive function training in children, there are some limitations to this study that should be acknowledged. First, one methodological shortcoming is that the baseline performance levels varied on the flanker task between groups. Specifically, the control group had a shorter overall RT on the pretest of the flanker task compared to the training group. Training benefits were the greatest when baseline performance was the lowest (Zuber et al., 2023), so the training effects of the training group in the current study may have been exaggerated compared to the control group. Nevertheless, we believe that simply implementing a similar program with fidelity can be effective, as the training results from longitudinal comparisons of the training groups are optimistic. Second, the intervention dose is still controversial. In this study, we did not find greater near- and far-transfer effects with the combined group. This is crucial because previous research has indicated that the dose might be associated with the cognitive training transfer effect (Schwaighofer et al., 2015), including training specific to early mathematics (Muñez et al., 2022). However, it is challenging to pinpoint the optimal dosage due to considerable variation in the duration of interventions across different studies (Nelson & McMaster, 2019). Third, previous studies have indicated that there are certain discrepancies between laboratory-based cognitive tests and daily cognitive tests in terms of their intended relevance and applicability (Bielak et al., 2017). Whether the transfer effects discovered in this study can be effectively observed in real-life daily situations still requires further verification through rigorous empirical research. Fourth, as mentioned above, with increasing age, executive functions become more specialized and independent, leading to a reduction in the shared neural mechanisms underlying updating, inhibition, and switching (Hartung et al., 2020; F. Xu et al., 2013). Given this, conducting combined training for young children in the future may enable us to discover far-transfer effects. Finally, although this study initially observed changes in the ERP results of the combined group after the training, it did not conduct a follow up to examine whether the benefits brought about by the training persisted over time. Previous research has shown that the intervention effects tend to diminish over time (Bailey et al., 2017). Future research can further optimize training programs, conduct long-term follow-up studies, and integrate brain structural imaging techniques to gain a more comprehensive and in-depth understanding of the relationship between children’s executive function performance and cortical changes.

## 5. Conclusions

We found that in the single-training group, employing the 2-back task, a significant near-transfer effect on the updating component was produced, while in the combined-training group, employing both 2-back and arrow flanker tasks, clear near-transfer effects on both updating and inhibition components were produced. Far-transfer effects on switching and fluid intelligence were not produced in either training group. Relative to the single-training group, the combined-training group did not exhibit a much greater transfer effect. These results show that the extent of transfer depends on cognitive component overlap between the training and transfer tasks. The electrophysiological results showed a significant reduction in N2 amplitude and a significant increase in P300 amplitude after combined training, suggesting that participants gained enhanced inhibition ability and improved updating capacity after training. From the perspective of near-transfer effects, the efficiency of the combined training is better than that of single training. However, further studies are needed to investigate which components targeted by combined training will produce greater far-transfer effects. Future studies should consider the effects of both the intensity and duration of training on far-transfer effects.

## Figures and Tables

**Table 1:** Mean d′ scores for the three groups at the pretest and posttest (standard deviation of the mean).

Training Group	Pretest		Posttest	
	*M* (*SD*)	Range	*M* (*SD*)	Range
control	1.42 (0.81)	0.13–2.35	1.63 (0.79)	−0.41–3.45
single	1.14 (0.69)	0.28–3.45	2.00 (0.96)	−0.20–3.56
combined	1.44 (0.43)	0.13–3.45	2.26 (0.59)	−0.41–3.73

**Table 2:** Mean reaction times (ms) for congruent and incongruent trials for the three groups at pretest and posttest (standard deviation of the mean).

Training Group	Pretest				Posttest			
	Congruent	Range	Incongruent	Range	Congruent	Rang	Incongruent	Range
control	477 (84)	380–695	478 (126)	431–974	451 (59)	371–606	450 (91)	415–816
single	512 (56)	423–667	663 (123)	521–1018	500 (85)	401–771	590 (101)	472–902
combined	515 (81)	389–690	693 (136)	442–934	455 (65)	365–563	554 (99)	431–773

**Table 3:** The mean reaction times (ms) and accuracy for the repeating and switching trials for the three groups at pretest and posttest (standard error of the mean).

Training Group	Pretest				Posttest			
	Repeating		Switching		Repeating		Switching	
	RT	ACC	RT	ACC	RT	ACC	RT	ACC
control	813 (27)	0.80 (0.03)	926 (30)	0.63 (0.03)	790 (21)	0.83 (0.03)	875 (25)	0.71 (0.02)
single	846 (21)	0.74 (0.03)	908 (25)	0.61 (0.03)	731 (31)	0.75 (0.03)	812 (40)	0.65 (0.03)
combined	866 (30)	0.80 (0.04)	1005 (42)	0.59 (0.04)	807 (33)	0.82 (0.03)	935 (39)	0.67 (0.03)

*Note:* RT, reaction time; ACC, accuracy.

**Table 4:** The mean and standard deviations of Raven’s standard progressive matrix Z-scores for the three groups.

Training Group	Pretest		Posttest		Tracking Test	
	*M* (*SD*)	Range	*M* (*SD*)	Range	*M* (*SD*)	Range
control	−0.35 (0.46)	−0.92–0.50	−0.22 (0.42)	−0.92–0.50	−0.24 (0.69)	−1.34–1.13
single	−0.30 (0.41)	−1–0.36	−0.38 (0.37)	−1.17–0.31	−0.20 (0.46)	−1–0.64
combined	−0.38 (0.60)	−1.56–0.67	−0.22 (0.50)	−1.08–0.67	−0.22 (0.53)	−0.95–1.23

**Table 5:** Mean d′ scores for the two groups at pretest and posttest (standard deviation of the mean).

Training Group	Pretest		Posttest	
	*M* (*SD*)	Range	*M* (*SD*)	Range
control	1.57 (0.64)	0.13–2.42	1.69 (0.56)	0.59–3.06
combined	1.46 (0.58)	0.38–2.47	2.12 (0.42)	1.56–3.11

**Table 6:** The mean reaction times (ms) for the congruent and incongruent trials for the two groups at pretest and posttest (standard deviation of the mean).

Training Group	Pretest				Posttest			
	Congruent	Range	Incongruent	Range	Congruent	Range	Incongruent	Range
control	468 (100)	383–833	596 (116)	461–914	456 (83)	351–701	546 (102)	377–797
combined	503 (83)	388–690	669 (153)	442–934	452 (62)	365–560	533 (95)	427–773

## Data Availability

The datasets used and analyzed during the current study are available from the corresponding author on reasonable request.
